# Recent Advances of Activatable Molecular Probes Based on Semiconducting Polymer Nanoparticles in Sensing and Imaging

**DOI:** 10.1002/advs.201600481

**Published:** 2017-02-09

**Authors:** Yan Lyu, Kanyi Pu

**Affiliations:** ^1^ School of Chemical and Biomedical EngineeringNanyang Technological University70 Nanyang DriveSingapore637457

**Keywords:** activatable probes, nanoparticles, photoacoustic imaging, semiconducting polymers, sensing

## Abstract

Molecular probes that change their signals in response to the target of interest have a critical role in fundamental biology and medicine. Semiconducting polymer nanoparticles (SPNs) have recently emerged as a new generation of purely organic photonic nanoagents with desirable properties for biological applications. In particular, tunable optical properties of SPNs allow them to be developed into photoluminescence, chemiluminescence, and photoacoustic probes, wherein SPNs usually serve as the energy donor and internal reference for luminescence and photoacoustic probes, respectively. Moreover, facile surface modification and intraparticle engineering provide the versatility to make them responsive to various biologically and pathologically important substances and indexes including small‐molecule mediators, proteins, pH and temperature. This article focuses on recent advances in the development of SPN‐based activatable molecular probes for sensing and imaging. The designs and applications of these probes are discussed in details, and the present challenges to further advance them into life science are also analyzed.

## 1 Introduction

Understanding and imaging biological and pathological processes are important for early diagnosis and therapy.^1^ However, chemical mediators in signal transduction^2^ and diseases hallmarks^3^ in pathological conditions are usually in low quantity and have sophisticated biological functions. The diversity and complexity of the microenvironment of living organisms create additional challenges to detect the target of interest.^4^ Molecular probes have been widely used to detect biomarkers and molecular events in living organisms, which can be divided to “always on” and activatable probes.^5^ “Always on” probes develop contrast signals through accumulation and they do not change signals upon interaction with the target of interest.^6^ In contrast, activatable probes, such as molecular beacons or optical switches, are in the “off” state at the beginning and only can be activated by the target to send out signals (**Figure** [qv: **1**]a).^5^, ^7^ Thereby, as compared with the conventional “always on” probes, activatable molecular probes have higher signal‐to‐noise ratio and lower detection of limit, permitting sensitive and real‐time detection of biomarkers in living organisms.^8^

**Figure 1:**
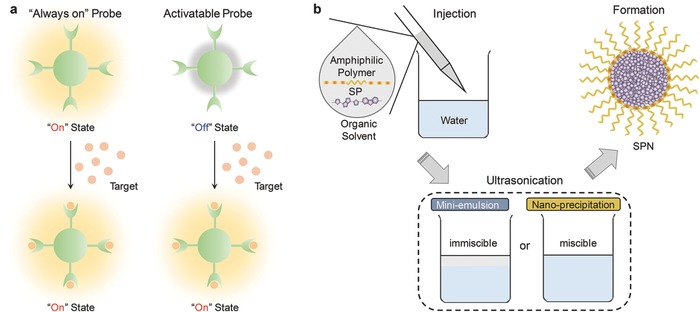
a) Illustration of “always on” and activatable probes. “Always on” probes develop signal contrast through accumulation in disease site and they do not change signals upon interaction with the target of interest; in contrast, activatable probes are in “off” state at the beginning and turned “on” in the presence of the target. b) Illustration of the preparation methods of SPNs: mini‐emulsion and nano‐precipitation. Both methods need to dissolve SPs and amphiphilic polymers (optional) in an organic solvent first and then the mixture is injected to the water to form nanoparticles. The difference is that the organic solvent used for mini‐emulsion is immiscible with water but miscible for nano‐precipitation.

Activatable molecular probes can often correlate their signals with the quantity and activity of biomarkers. For instance, enzyme activity can be translated to fluorescence signals through enzymatic cleavage that turns the probe from the “off” state to the “on” state.^9^ Although radioactive^10^ and magnetic signals^11^ are also feasible to be used as the readout signals, the optical signals are more widely used due to the easy implementation and relatively low‐cost instruments.[[qv: 7a,12]] Along with real‐time detection capability, the broad emission range of optical materials ranging from visible spectrum to near infrared (NIR) region^13^ makes it feasible to achieve multiple spectral imaging. Until now, fluorophores,[[qv: 7b,14]] genetically engineered fluorescent proteins,^15^ quantum dots,^16^ and gold nanoclusters[[qv: 7b]] have all been developed into fluorescent activatable probes.

Semiconducting polymer nanoparticles (SPNs) have formed a new class of photonic nanomaterials for the development of activatable molecular probes.^17^ The major components of SPNs are semiconducting polymers (SPs), which are polymers with π‐electron delocalized backbones.^18^ Thus, the optical properties of SPNs are mainly determined by the molecular structures of SPs.^19^ Because the band gaps of SPs can be turned by the monomers used for the polymerization,^20^ SPNs have the structural versatility to fulfill different imaging tasks. The common ways to prepare SPNs are mini‐emulsion and nanoprecipitation (Figure 1b). The preparation methods have important effect on the diameters of SPNs. Generally, SPNs prepared by mini‐emulsion are larger (40 to 500 nm)^21^ than those prepared by nanoprecipitation (5 to 50 nm).^22^ Both methods need to dissolve SPs and amphiphilic polymers (optional) in organic solvent prior to injection into water to form nanoparticles. The difference is that the organic solvent used for mini‐emulsion is immiscible with water but miscible for nanoprecipitation.

Distinct from inorganic nanoparticles such as quantum dots and gold nanoclusters, SPNs are composed of benign organic ingredients including hydrophobic SPs and amphiphilic polymer matrixes (optional).^22^, ^23^ SPNs thereby avoid the issue of heavy metal ion induced toxicity and have good biocompatibility.^24^ Additionally, SPNs generally have higher absorption coefficients and better photostability compared to small‐molecule dyes.^25^ This also stands when comparing SPNs with supramolecular nanoparticles, as they are generally assembled from small‐molecule dyes.^26^ Facile PEGylation (where PEG is polyethylene glycol) generally endows SPNs with good biodistribution, allowing them to act as whole‐body imaging agents to detect the target of interest in living animals after systemic administration.^27^ With all these advantages, SPNs have been widely used in cells imaging,[[qv: 27a,d]] activated cell sorting,^28^ sensing of chemical mediators,[[qv: 17b,c]] tumor imaging[[qv: 27e,29]] hemodynamic imaging,[[qv: 27g]] and optogenetics.^30^

There are some review articles summarizing the synthesis and applications of SPNs.[[qv: 17d,22a,24a,31]] This review focuses on the recent advances in the use of SPN‐based activatable probes for sensing and molecular imaging. We discuss the chemistry of these organic nanoprobes in terms of different photonic imaging modalities along with their sensing and imaging applications. The potential challenges and the perspectives are also analyzed.

## 2 Photoluminescence

### 2.1 Gaseous Molecule Sensing

#### 2.1.1 Oxygen

Oxygen is an important physiological index and the deprivation of oxygen is usually related to some pathological conditions such as tumor growth,^32^ diabetic retinopathy^33^ and rheumatoid arthritis.^34^ SPNs have been employed for oxygen sensing by taking advantage of oxygen‐sensitive phosphorescence of organometallic dyes.^35^

**Figure 2:**
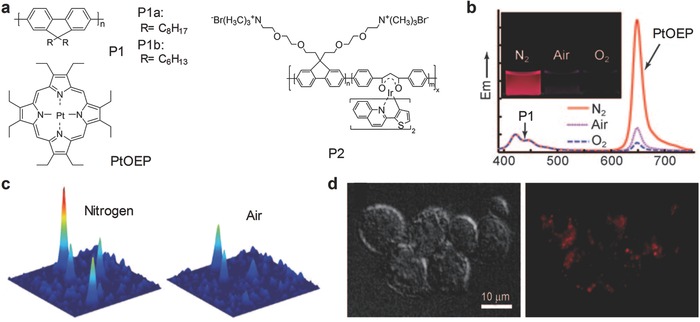
SPN‐based activatable probes for photoluminescence ratiometric sensing of oxygen. a) Chemical structures of SPs and an organic dye (PtOEP) used for preparation of SPN‐based activatable probes for oxygen detection. b) Oxygen‐dependent emission spectra of the SPN‐P1b probe (excitation at 350 nm). The inset showed the photographs of the SPN‐P1b aqueous solution saturated with nitrogen, air and oxygen, respectively, under a UV lamp irradiation. c) Single‐particle phosphorescence images of the SPN‐P1b probe immobilized on coverslips under nitrogen or air atmosphere. d) DIC and phosphorescence images indicated the uptake of the SPN‐P1 probe by J774A1 cells. Reproduced with permission.^25^ Copyright 2009, Wiley‐VCH Verlag GmbH & Co. KGaA, Weinheim.

The SPN probes mainly composed of poly(9,9‐dioctylfluorene) (PFO) (P1a, **Figure** [qv: **2**]a) or poly(9,9‐dihexylfluorene) (PDHF) (P1b, Figure 2a) and doped with an oxygen sensitive dye, platinum(II) octaethylporphine (PtOEP, Figure 2a), were used for molecular oxygen sensing.^25^ With increased oxygen concentration, the luminescence at 650 nm caused by fluorescence resonance energy transfer (FRET) from the SP to the dye were gradually quenched, while the emission of P1 at 420 nm remained nearly unchanged. Thus, the molecular oxygen was able to be ratiometrically detected (Figure 2b). The SPN‐P1b probe was stable and its phosphorescence could be recovered after being exposed to nitrogen without apparent photobleaching. Additionally, the SPN‐P1b probe was able to respond to both dissolved and atmosphere oxygen (Figure 2b,c). Because the cellular uptake of the SPN‐P1 probe was validated (Figure 2d), this probe is promising for detection of oxygen in living cells and tissues.

Similarly, a phosphorescent SP containing Ir(III) complex (P2) was synthesized and transformed into the nanoparticles for naked‐eye detection of oxygen in aqueous solution (Figure 2a).^36^ The intrapolymer FRET existed within SPN‐P2 probe, leading to the phosphorescence from the Ir(III) complex that was sensitive to oxygen. The oxygen quenching efficiency, an indication of sensitivity, was measured to be 96.7%, similar to that of the SPN‐P1 probe (≈95%). In addition, the SPN‐P2 probe was found to produce singlet oxygen under irradiation at 488 nm, showing its potential for photodynamic therapy (PDT).

#### 2.1.2 Reactive Oxygen and Nitrogen Species

Reactive oxygen and nitrogen species (RONS) are the chemically active species containing oxygen or nitrogen. Although RONS are usually the byproducts of natural metabolism^37^ that play important roles in homeostasis^38^ and signals transduction,^39^ the dramatically increased amount of RONS under extreme environment, also known as oxidative stress, are the hallmarks of many diseases,^40^ such as bacterial infection,^41^ cancer,^42^ cardiovascular disease^43^ and arthritis.^44^ Despite the importance of RONS in biology and medicine, activatable probes capable of detecting them in vivo are still limited.

**Figure 3:**
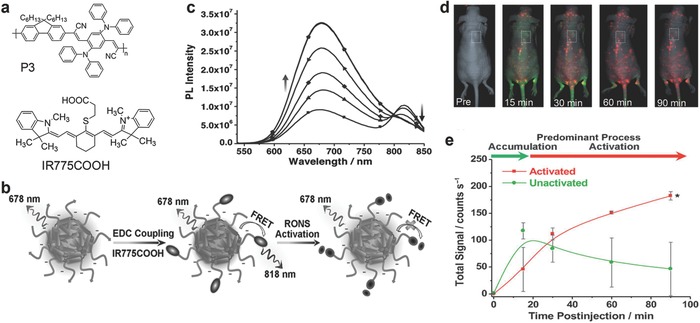
SPN‐based activatable probes for fluorescence ratiometric imaging of reactive oxygen and nitrogen species (RONS). a) The chemical structure of P3 and an organic dye (IR775COOH) used for preparation of the SPN‐P3 probe for RONS detection. b) Schematic of the preparation and RONS sensing mechanism of the SPN‐P3 probe. c) Fluorescence spectra of the SPN‐P3 probe upon addition of ONOO^−^. Fluorescence images d) and signal quantification e) of living mice with spontaneous systemic *C. bovis* bacterial infection injected with the SPN‐P3 probe through tail vein injection. Pseudo‐colors represent the activated (red) and inactivated states (green) of the probe. *Statistically significant difference change in the fluorescence intensities between the SPN‐P3 probe in the inactivated and activated state (*n* = 4, P < 0.05). Reproduced with permission.[[qv: 17a]] Copyright 2013, Wiley‐VCH Verlag GmbH & Co. KGaA, Weinheim.

The fluorescence ratiometric probe based on a NIR‐emissive SP, poly[9,9′‐dihexyl‐2,7‐bis(1‐cyanovinylene)fluorenylene‐*alt*‐*co*‐2,5‐bis(*N*, *N*‐diphenylamino)‐1,4‐phenylene] (PCFDP) (P3, **Figure** [qv: **3**]a), was developed for RONS imaging.[[qv: 17a]] The SPN‐P3 nanoparticles were conjugated with a RONS sensitive cyanine derivative (IR775COOH) through a carbodiimide‐activated coupling reaction (Figure 3a). Due to FRET (Figure 3b), the SPN‐P3 probe had a dual emission peak at 678 and 818 nm corresponding to P3 and IR775COOH, respectively. In the presence of RONS, IR775COOH was cleaved and the FRET process was thus inhibited, leading to probe activation. Thereby, the emission from P3 (678 nm) was gradually recovered with the emission decrease at 818 nm, allowing for ratiometric fluorescence imaging of RONS (Figure 3c). The SPN‐P3 probe was responsive to peroxynitrite (ONOO^−^), hypochlorite (ClO^−^) and hydroxyl radical (·OH) but not to hydrogen peroxide (H_2_O_2_) and other RONS. The probe was used to image elevated production of RONS in RAW264.7 cells and acute inflammation mouse model treated with bacterial cell wall lipopolysaccharide (LPS) and phorbol 12‐myristate 13‐acetate (PMA). The ratiometric signals allowed one to assign the states of the probe using pseudo colors: green and red for inactivated and activated probes, respectively. The probe was also tested for real‐time imaging of RONS in mice infected with bacteria *Corynebacterium bovis* (*C. bovis*) (Figure 3d,e). After systemic administration, the probe accumulated in the bacterial infection sites (pseudo‐green) within 20 min post‐injection, which was attributed to the enhanced permeability and retention (EPR) effect. The probes were gradually activated (pseudo‐red) by RONS produced in infection regions and reached complete activation at 60 min post‐injection.

### 2.2 Ion Detection

**Figure 4:**
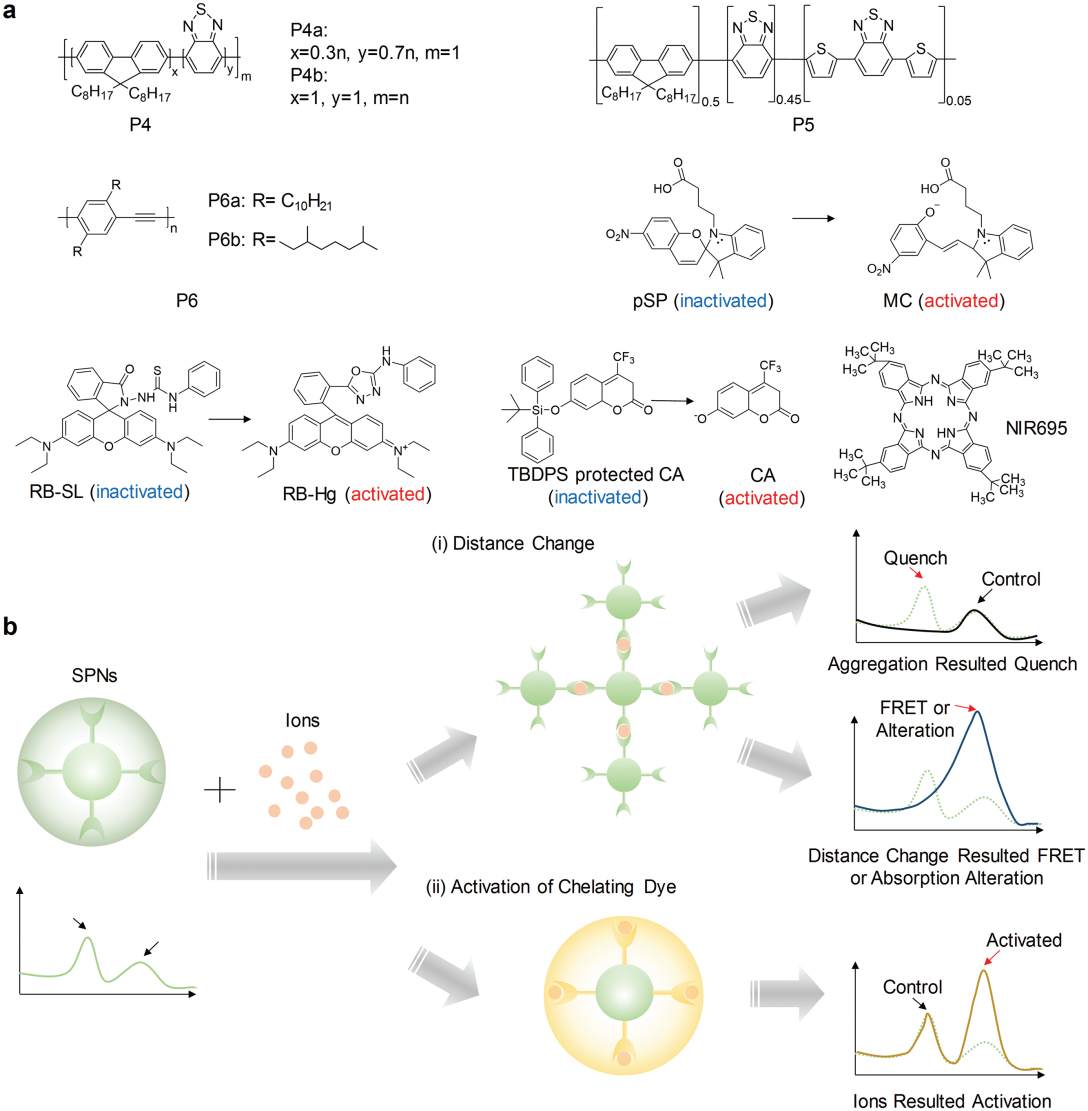
SPN‐based activatable probes for fluorescence sensing of ions. a) Chemical structures of SPs and organic dyes used for preparation of SPN‐based probes for ion detection. b) Common mechanisms used in SPN‐based probes for ion detection. i) SPNs chelate with ions to result in the distances change among nanoparticles. In one situation, the fluorescence will be gradually quenched due to crosslinking‐caused nanoparticle aggregation; in the other situation, the shortened distance will promote FRET or alter the absorption because of the distortion of the conjugated chains. ii) The sensing component within SPNs can be activated by the chelation with ions, resulting in a new emission peak.

The SPs in **Figure** [qv: **4**]a, including poly[(9,9‐dioctylfluorenyl‐2,7‐diyl)‐*co*‐1,4‐benzo‐{2,1′‐3}‐thiadiazole] (PFBT) (P4), poly[(9,9‐dioctylfluorene)‐*co*‐2,1,3‐benzothiadiazole‐*co*‐4,7‐di(thiophen‐2‐yl)‐2,1,3‐benzothiadiazole] (PFBT‐DBT) (P5) and poly(2,5‐di(3′,7′‐dimethyloctyl) phenylene‐1,4‐ethynylene) (PPE) (P6), were used for the construction of activatable probes for detection of various ions. The probes were capable to specifically chelate with ions. The chelating process can induce the distances change among particles or transform the sensing component from non‐fluorescence to fluorescence state, ultimately changing the optical properties of SPNs (Figure 4b). There are two potential effects when the interparticle distance is changed: i) the fluorescence will be gradually quenched as a result of chelation‐induced aggregation of SPNs; ii) FRET will be enhanced due to the shortened donor–acceptor distance, or absorption change will be induced because of the distortion of the conjugated chains. The distance change and the states transformation could also work together to respond to specific ions.

#### 2.2.1 Copper and Iron Ions

Copper (Cu^2+^) and iron (Fe^2+^) ions belong to the most abundant transition metal ions in human body, but the amount need to be accurately controlled to avoid toxicity.^45^ There are ongoing efforts to prepare sensitive probes to detect these ions and the P4b‐based probe is one of the examples. P4b was co‐precipitated with poly(styrene‐*co*‐maleic anhydride) (PSMA) (20%) to form the SPN‐P4b probe with carboxyl groups on the surface.^46^ In the presence of Cu^2+^ or Fe^2+^, the SPN‐P4b probe aggregated because of interparticle crosslinking upon coordination with metal ions, resulting in fluorescence quench at 540 nm. Moreover, a ratiometric probe was created using the carboxyl‐free SPNs with the stable emission at 623 nm as the internal standard. The probe exhibited good linearity and high selectivity to Cu^2+^ and Fe^2+^. Further differentiation and individual quantification of Cu^2+^ and Fe^2+^ were achieved with the help of the Cu^2+^‐selective recovery capability of SPN‐P4b.

Another probe for specific detection of Cu^2+^ was developed using two different SPs. First, P4a and P6a were doped with a photoswitchable spiropyran (pSP, Figure 4a). Then the resulted two SPNs, pSP‐SPN‐P4a and pSP‐SPN‐P6a, were mixed to form the probe.^47^ After UV irradiation, pSP was activated to the open merocyanine (MC) form with the capability of chelating Cu^2+^ attributed to the negatively charged phenolate oxygen atoms (Figure 4a). This shortened the distance between pSP‐SPN‐P4a and pSP‐SPN‐P6a, and thus promoted FRET, permitting the detection of Cu^2+^ in the physiological range. Additionally, the probe could be renewed under the acidic environment with the irradiation of white light that transferred MC back to pSP and released Cu^2+^.

#### 2.2.2 Mercury Ions

With the well‐established toxic effects of mercury ions (Hg^2+^) on health and ecosystems, it is essential to detect extremely low concentration of Hg^2+^.^48^ A Hg^2+^ responsive SPN probe was developed based on P4b and a rhodamine spirolactam dye (RB‐SL) (Figure 4a).^49^ RB‐SL can be turned into RB‐Hg by Hg^2+^. Thus, in the presence of Hg^2+^, the emission of RB‐Hg at 590 nm was significantly enhanced along with the decreased emission of P4b at 537 nm due to FRET process. The probe was specific to Hg^2+^, and the quantification was achieved using the ratiometric fluorescence signals (*I*_590_/*I*_537_), showing the detection of limit as low as 0.7 parts per billion (ppb).

#### 2.2.3 Silver Ions

The contamination of surface water and adverse effect on human caused by silver are of vital concern.^50^ Thus, there is an urgent need to detect silver ions (Ag^+^) with high selectivity in aqueous milieu and biological systems. An activatable probe for Ag^+^ sensing was developed by encapsulating P4 with sulfonated polystyrene‐*block*‐poly(ethylene‐*ran*‐butylene)‐*block*‐polystyrene (PS‐SO_3_H).^51^ Benzothiadiazole of P4 not only provided the read‐out signals, but also played the role of specific chelating with Ag^+^. Meanwhile, the amphiphilic nature of PS‐SO_3_H partially took part in the chelating process. Thus, the probe showed specific aggregation‐induced fluorescence quenching towards Ag^+^ but not for other 12 metal cations, and its fluorescence could be recovered to the original state by adding NaCl to sequestrate Ag^+^.

#### 2.2.4 Fluoride Ions

Fluoride ions (F^−^) are one of the most important anions that are closely related to nerve gasses and numerous human diseases.^52^ A SPN‐based F^−^ probe was developed by blending P6b with nonfluorescent *tert*‐butyldiphenylsilyl (TBDPS)‐protected 7‐hydroxy‐4‐tri‐fluoromethyl coumarin (CA) (Figure 4a).^53^ The specific reaction between F^−^ and TBDPS led to the deprotected CA and thus turned on its fluorescence at 520 nm. Upon adding F^−^, CA was formed and because of efficient FRET, the fluorescence at 520 nm was significantly increased with the decreased emission of P6b (440 nm). Thus, the ratiometric fluorescence signals (I_520_/I_440_) towards F^−^ were achieved in the linear dynamic range of 0 to 160 μM, almost unaffected by other anions. Despite the capability of detecting F^−^, the short emission wavelength limited this probe to in vitro applications.

#### 2.2.5 Lead Ions

Lead, which is one of the most abundant and hazardous heavy metal elements in the environment, has been proven to cause serious health problems.^54^ However, detection of lead ions (Pb^2+^) usually requires sophisticated instruments and complicated sample pretreatment processes. An easily prepared SPN‐based probe for Pb^2+^ detection was developed by encapsulating P5 and a dye NIR695 (Figure 4a) with the carboxyl‐ and 15‐crown‐5‐functionalized polydiacetylenes (PDAs) mixture.^55^ The PDAs chelated Pb^2+^, shortened or partially distorted the conjugated system, and consequently led to chromatic transition from blue to red. Meanwhile, the NIR dye leached out, resulting in the disruption in FRET process and in turn changing the ratiometric fluorescence signals (*I*_650_/*I*_715_). The dual colorimetric and fluorescent probe was also developed into test strips for Pb^2+^ sensing.

### 2.3 pH Sensing

pH is regarded as an important parameter in physiological process, as it can directly alter the configuration^56^ and activity^57^ of biomolecules. The abnormal pH value disturbs physical homeostasis, and thus is associated with many diseases, such as inflammation,^58^ cancer,^59^ cardiac ischemia,^60^ and Alzheimer's disease.^61^ Thereby, sensing pH is important for life science.

Chiu's group designed a SPN probe based on P6a and a pH‐sensitive fluorescein (**Figure** [qv: **5**]a).^62^ The FRET between P6a and the fluorescein dye occurred, leading to two emission peaks: the dye emission at 513 nm that was responsive to pH and the other from P6a at 440 nm that was inert to pH. Thus, the SPN‐P6a probe could ratiometrically measure pH from 5 to 8, fulfilling the general requirements for cellular studies.

**Figure 5:**
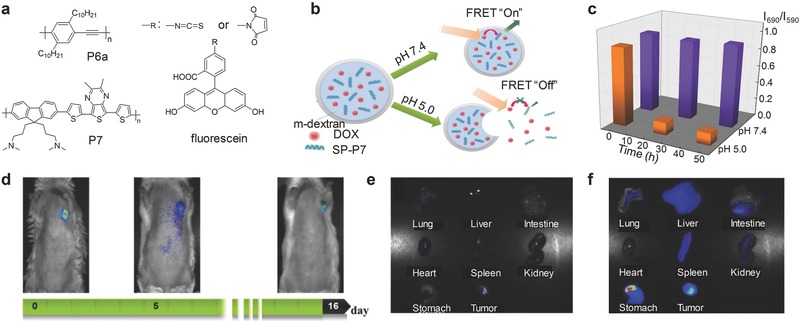
SPN‐based activatable probes for fluorescence sensing of pH. a) Chemical structures of SPs and the organic dyes used for preparation of SPN‐based probes for pH detection. b) Schematic illustration of signals alteration of the SPN‐P7 probe and its capability of controlling the drug release responsive to different pH. c) The ratiometric fluorescence signals (*I*_690_/*I*_590_) from the SPN‐P7 probe as the function of time under acidic or physiological conditions. Excitation at 480 nm. d) NIR images of subcutaneous tumor bearing mouse following local injection with the SPN‐P7 probe. Excitation at 455 nm. Ex vivo NIR fluorescence imaging on major organs of mice with excitation at e) 455 and f) 595 nm. Reproduced with permission.^63^ Copyright 2009, Royal Society of Chemistry.

Similarly, another SPN‐based probe for pH sensing was prepared using poly[9,9‐bis(N,N‐dimethylpropan‐1‐amino)‐2,7‐fluorene‐*alt*‐5,7‐bis(thiophen‐2‐yl)‐2,3‐dimethylthieno[3,4‐b]pyrazine] (BTTPF) (P7, Figure 5a) as the matrix.^63^ In addition to pH sensing, the SPN‐P7 probe was able to load drug and control its release by responding to pH. The pH‐sensitive ability of the probe resulted from the pendant acetal modified dextran (m‐dextran) used to encapsulate doxorubicin (DOX) and P7 (Figure 5b). In mildly acidic environment, m‐dextran was hydrolyzed, resulting in the dissociation of SPNs. Due to the separation of P7 and DOX, the FRET process was weakened. Thus, the P7 emission at 690 nm decreased and the DOX fluorescence was recovered (Figure 5c). In addition to pH sensing responsive capability, the NIR fluorescence of the SPN‐P7 probe allowed to track the drug release process in vivo. After local injection, the NIR fluorescence was longitudinally recorded within 16 days and showed significant decrease due to the drug release (Figure 5d), consistent with the bioditribution ex vivo (Figure 5e,f).

### 2.4 Temperature Sensing

Temperature is a fundamental parameter in biosystems because it directly affects enzyme activity,^64^ gene expression,^65^ and biological reaction equilibrium,^66^ all of which play important roles in metabolism to keep organisms alive. The abnormal temperature is related to disease conditions, such as cancer.^67^ However, accurate measurement of the localized temperature on the microscale remains a problem. The tunable cellular uptake of SPNs makes them suitable for developing non‐invasive and localized probes for temperature sensing in cells.

**Figure 6:**
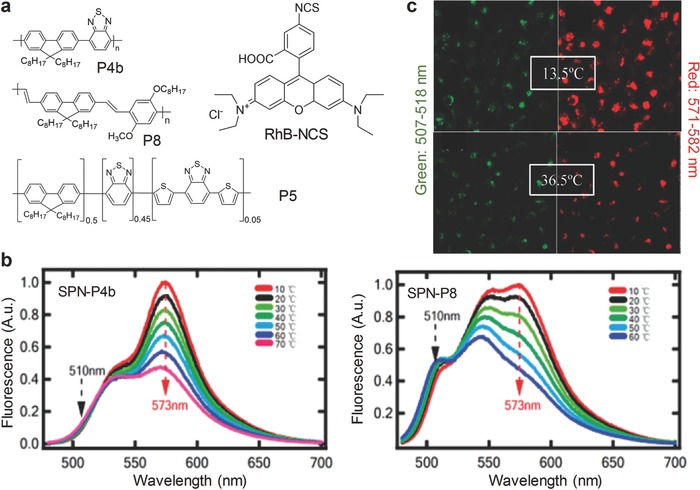
SPN‐based activatable probes for fluorescence sensing of temperature. a) Chemical structures of SPs and the organic dye (RhB‐NCS) used for preparation of SPN‐based probes for temperature detection. b) Fluorescence spectra of the SPN‐P4b and SPN‐P8 probes at different temperatures. Excitation at 450 nm. c) Confocal laser microscopy images of HeLa cells incubated with the SPN‐P4b probe at 13.5 or 36.5 °C. Excitation at 458 nm. Emission at 507–518 nm was indicated in pseudo green and 571–582 nm was indicated in pseudo red. Reproduced with permission.^68^ Copyright 2011, American Chemical Society.

The temperature‐sensitive probes were developed based on P4b or poly[{9,9‐dioctyl‐2,7‐divinylene fluorenylene}‐*alt*‐*co*‐{2‐methoxy‐ 5‐(2‐ethylhexyloxy)‐1,4‐phenylene}] (PFPV) (P8) (**Figure** [qv: **6**]a).^68^ After doping with a temperature‐sensitive dye Rhodamine B (RhB) (Figure 6a), the SPN‐P4b or SPN‐P8 probe was able to ratiometrically measure temperature. The efficient FRET was achieved from P4b or P8 to RhB and the temperature‐dependent peak at 573 nm appeared which decreased with increasing temperature (Figure 6b). Taking unchangeable signals at 510 nm as the internal reference, SPN‐P4b probe could quantify the temperatures in Hela cells through ratiometric fluorescence signals (*I*_507–518_/*I*_571–582_), which were in good accordance with the spiking values measured by thermocouple without interference from the bio‐environment (Figure 6c).

Another probe for temperature sensing was realized using PDAs to encapsulate P5 (Figure 6a) and a dye NIR695 (Figure 4a).^69^ Once the temperature increased, the FRET system was inhibited due to the disturbance of PDA accompanied by subsequent bleaching out of the dye. The capability of this probe for temperature sensing was demonstrated in the test papers prepared with the SPN‐P5 solution. However, it might be difficult for these test papers to detect the subtle temperature changes.

### 2.5 Enzyme

**Figure 7:**
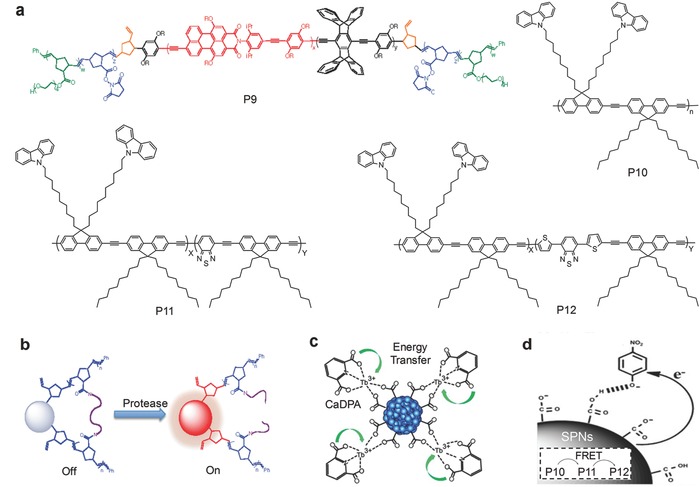
SPN‐based activatable probes for fluorescence sensing of other targets. a) Chemical structures of SPs used for preparation of SPN‐based probes. Reproduced with permission.^71^ Copyright 2012, American Chemical Society. b) The mechanism for protease detection. The fluorescence was initially quenched by the peptide crosslinking and recovered after digestion by protease. Reproduced with permission.^71^ Copyright 2012, American Chemical Society. c) The mechanism for CaDPA detection. Coordination with CaDPA resulted increased fluorescence of lanthanide ions. Reproduced with permission.^73^ Copyright 2013, American Chemical Society. d) The mechanism of nitroaromatics detection. In the presence of nitroaromatics, PET from the electron‐rich SPNs to the electron‐deficient nitroaromatics (such as the model compound, nitrophenol) occurred to quench the fluorescence of the SPNs. Reproduced with permission.^75^ Copyright 2015, Royal Society of Chemistry.

Considering the overexpression of proteases in cancer cell lines, the alteration of proteolytic activities is potential to be developed as an early marker for cancer diagnosis.^70^ Inspired by the potential to be used in in vivo imaging, a copolymer poly(phenylene ethynylene) (PPE)‐succinimidyl ester (NHS)‐tetraethylene glycol (TEG) (P9, **Figure** [qv: **7**]a) based SPNs were developed, which had the emission at 604 nm through intramolecular FRET.^71^ It was capable of detecting protease activity with fluorescence turn‐on (Figure 7b). Initially, the fluorescence was significantly quenched by the cross‐link with a trypsin identified peptide. After digested by trypsin, the swelling‐like (strain‐release) mechanism resulted in the fluorescence recovery, which was proved by 15‐ and 12‐fold fluorescence increase at 454 and 604 nm, respectively.

### 2.6 Calcium Dipicolinate

Calcium dipicolinate (CaDPA) is a biomarker for bacterial spores, which are resistant to severe environment and can germinate after elimination of extreme pressure.^72^ Therefore, in public health, it is critical to develop probes for fast detection and quantification of CaDPA to evaluate the potential of bacteria‐induced infection. Thus, a SPN activatable probe based on P1a (Figure 2a) chelated with lanthanide ions was developed to detect CaDPA.^73^ In the presence of CaDPA, the luminescence of lanthanide ions significantly was sensitized upon CaDPA chelation (Figure 7c), while the emission from P1a remained unchanged and served as the internal reference.

### 2.7 Nitroaromatics

Nitroaromatics, as the raw ingredients in explosives, threaten human safety and health and it is therefore important to develop molecular probes to detect them.^74^ Three different SPs, P10, P11, and P12, were encapsulated together to achieve emission at 614 nm with effective FRET (Figure 7a,d).^75^ Upon encountering nitroaromatics, photoinduced electron transfer (PET) from electron‐rich SPNs to electron‐deficient nitroaromatics (such as the model compound, nitrophenol) occurred to quench the fluorescence of SPNs (Figure 7d). Detection of nitroaromatics using SPNs was tested both in solutions and test trips.

## 3 Chemiluminescence

Chemiluminescence, i.e., the emission of light from chemical reactions, was widely used in the analysis of gas, inorganic/organic species and biomolecules.^76^ Compared with traditional detection methods, it significantly improves signal‐to‐noise ratio and reduces photodamage due to the removal of external light.

### 3.1 Multiplex Imaging of RONS

**Figure 8:**
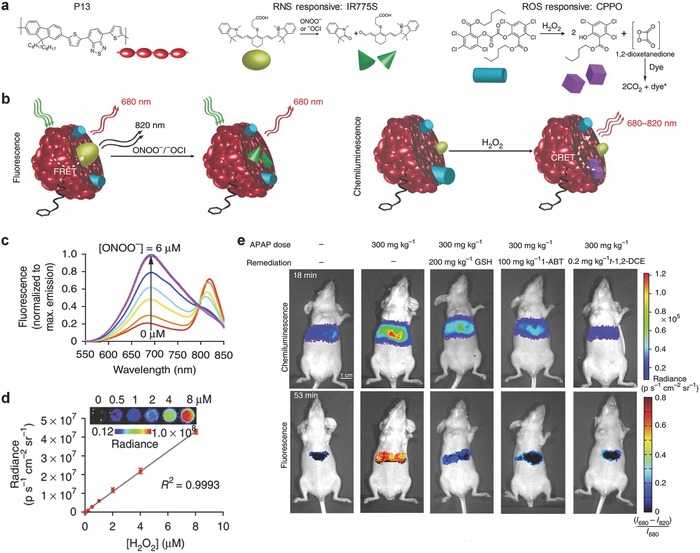
SPN‐based activatable probes for multiplex imaging of RONS. a) The chemical structure of P13, the organic NIR dye (IR775S) and the chemiluminescence substrate (CPPO) used for preparation of the SPN‐P13 probe for multiplex imaging of RONS. b) Illustration of the mechanism of simultaneous and differential detection of RNS (ONOO^−^ or ^−^OCl) and ROS (H_2_O_2_). In vitro detection of RNS (ONOO^−^) c) and ROS (H_2_O_2_) d) in 1×PBS solution. Representative images of mice receiving, from left to right, saline (‐), APAP intraperitoneally alone, and APAP with GSH, 1‐ABT or *t*‐1,2‐DCE, followed by intravenous injection of the SPN‐P13 probe. Reproduced with permission.[[qv: 17c]] Copyright 2014, Macmillan Publishers Limited, part of Springer Nature.

RONS can be used to predict hepatotoxicity for drug‐safety assays.^77^ The proof‐of‐concept application of multiplex imaging of RONS in vivo has been achieved using SPNs based on poly(2,7‐(9,9‐dioctylfluorene)‐*alt*‐4,7‐bis(thiophen‐2‐yl)benzo‐2,1,3‐thiadiazole) (PFODBT) (P13) (**Figure** [qv: **8**]a).[[qv: 17c]] In addition to the SP, the SPN‐P13 probe contained a ONOO^−^ responsive NIR dye IR775S (Figure 8a) and a H_2_O_2_ reactive chemiluminescent substrate, bis‐(2,4,5‐trichloro‐6‐(pentyloxycarbonyl)phenyl)oxalate (CPPO) (Figure 8a). This allowed the SPN‐P13 probe to simultaneously and differentially detect ONOO^−^ and H_2_O_2_ according to the fluorescence and chemiluminescene signals, respectively. In the presence of ONOO^−^, efficient FRET from P13 to IR775S was disrupted by ONOO^−^ because IR775S was oxidized by ONOO^−^. This resulted in the increased emission of P13 at 680 nm (Figure 8b,c) and the decreased emission of IR775S at 820 nm. In the presence of H_2_O_2_, the chemiluminescent reaction of CPPO was induced to produce photons as the product, leading to the luminescence from the probe without external light excitation (Figure 8b). The in vitro fluorescence and chemiluminescence signals could be correlated with the concentration of ONOO^−^ and H_2_O_2,_ respectively (Figure 8c,d). The capability of real‐time imaging of RONS was further tested in living mice suffered from hepatotoxicity (Figure 8e). Both the ratiometric fluorescence ((*I*_680_–I_820_)/*I*_680_) and chemiluminescence signals increased after challenging with the drugs such as the analgesic and anti‐pyretic acetaminophen (APAP) or the anti‐tuberculosis agent isoniazid (INH). The signals were reduced upon remediation with the RONS scavenger, glutathione (GSH), and the inhibition agents including 1‐aminobenzotriazole (1‐ABT) and *trans*‐1,2‐dichloroethylene (*t*‐1,2‐DCE). The SPN‐P13 probe was also used to conduct a mechanistic study of drug metabolism in living mice.

### 3.2 H_2_O_2_

**Figure 9:**
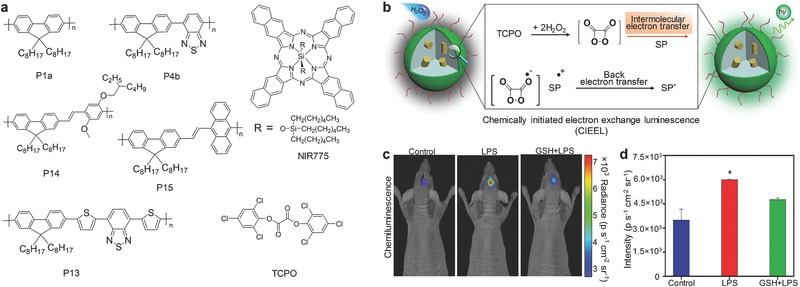
SPN‐based activatable probes for chemiluminescence imaging of H_2_O_2_. a) Chemical structures of SPs and the chemiluminescence substrate (TCPO) used for preparation of SPN‐based probes for chemiluminescence detection of H_2_O_2_. b) Illustration of the chemically initiated electron exchange luminescence (CIEEL) mechanism of SPNs. Representative chemiluminescence images (c) and quantification (d) of mice treated with saline, LPS or LPS with GSH, followed by intracerebral injection of the SPN‐P14 probe. *Statistically significant difference in the chemiluminescence intensities between LPS treated and untreated or GSH remediation mice (*n* = 3, *P* < 0.05). Reproduced with permission.^78^ Copyright 2016, American Chemical Society.

The chemiluminescence of SPNs can be further enhanced according to the mechanism of chemically initiated electron exchange luminescence (CIEEL).^78^ In CIEEL, the oxidation reaction occurs between the peroxalate bis(2,4,6‐trichlorophenyl) oxalate (TCPO) and H_2_O_2_, resulting in the production of the high energy intermediate (HEI), 1,2‐dioxetanedione. This intermediate first undergoes a reduction reaction by obtaining an electron from the SP, leading to production of the SP radical cation and the carbon dioxide radical anion. Then, back electron transfer occurs between the cation and the anion to produce the excited SPs, inducing the luminescence of SP. Thus, the key step that governs the chemiluminescence efficacy of SPNs is the intermolecular electron transfer from the SP to 1,2‐dioxetanedione. To amplify the chemiluminescence, different SPs were aligned with the HEI to facilitate the efficient electron transfer between the SP and the HEI to occur (**Figure** [qv: **9**]b). Among all SPs (Figure 9a), P14 had the highest chemiluminescence quantum yield (2.30 × 10^−2^ einsteins mol^−1^) because of the smallest gap between the highest occupied molecular orbital (HOMO) of P14 and the lowest unoccupied molecular orbital (LUMO) of 1,2‐dioxetanedione. Thus, SPN‐P14 efficiently detected H_2_O_2_ in vitro with the detection of limit as low as 5 nM. By doping SPN‐P14 with NIR775, the chemiluminescence wavelength was red‐shifted to the NIR region, and thus in vivo imaging of LPS‐induced neuroinflammation in mouse model was conducted. The signals for the LPS‐treated mice were 2.5‐fold higher than those for the control. GSH remediation led to reduced signals, confirming the ability of the SPN‐P14 probe to monitor the level of H_2_O_2_ in real‐time (Figure 9c,d).

### 3.3 Superoxide Anion

**Figure 10:**
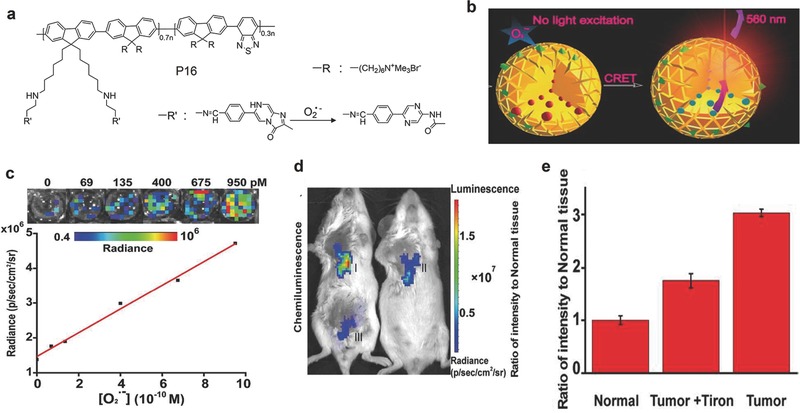
SPN‐based activatable probes for chemiluminescence imaging of superoxide anion (O_2_^•−^). a) The chemical structures of P16 used for preparation of the SPN‐P16 probe for chemiluminescence detection of O_2_^•−^. b) Illustration of the mechanism for detection of O_2_^•−^. c) The linear relationship between chemiluminescence signals of the SPN‐P16 probe and the concentration of O_2_^•−^. Inset, IVIS images of the SPN‐P16 probe in response to the indicated concentrations of O_2_^•−^ with open filter (570 ± 10 nm). Representative images of mice (d) and the quantification (e) of luminescence signals for tumor (I), tumor+Tiron (II) and normal (III) tissues followed by injection of the SPN‐P16 probe. Images (λ_em_ = 570 nm ± 10 nm) were acquired using an IVIS Lumina II at 30 s after local administration of the SPN‐P16 probe. Reproduced with permission.^80^ Copyright 2016, American Chemical Society.

As the primary ROS, superoxide anion (O_2_^•−^) exists at extremely low concentration in living system but acts as the early prediction for burst of many other radicals.^79^ Therefore, it is required to develop ultrasensitive probes for O_2_^•−^ detection. A copolymer (P16, **Figure** [qv: **10**]a), comprising a random copolymer of PF and PFBT as the main backbone and the imidazopyrazinone groups on the side chains, was transformed into the SPN‐P16 probe for O_2_^•−^ detection.^80^ Imidazopyrazinone as the chemiluminescence substrate could react with O_2_^•−^ to produce photons as the product, and the energy transfer eventually led to the luminescence from the SP backbone (Figure 10b). The SPN‐P16 probe could detect O_2_^•−^ down to picomole level and the produced signals showed good linearity with the concentration of O_2_^•−^ (Figure 10c). The SPN‐P16 probe was used to detect the generation of O_2_^•−^ in both LPS‐induced inflammation model and tumor model. After intratumoral injection of the SPN‐P16 probe, the chemiluminescence signals were detected from tumor tissues, which was three‐fold higher than that from normal tissues. After treatment with the typical superoxide scavenger Tiron, signals were reduced, indicating the decrease of O_2_^•−^ (Figures 10d,e). Thus, the SPN‐P16 probe was able to detect the variation of O_2_^•−^ in living mice.

## 4 Photoacoustic (PA) Imaging

PA imaging is a new non‐ionizing imaging technology that integrates optical excitation with ultrasonic detection based on the PA effect.^81^ It provides deeper tissue imaging penetration with higher spatial resolution as compared with traditional optical imaging techniques (e.g. fluorescence).^82^ Given its many key merits, PA imaging as one of the fastest‐growing molecular imaging technologies has been proved effective in tumor detection and molecular characterization.^83^ In addition, consistent with the principle for photothermal therapy (PTT) that requires efficient conversion of photon energy into heat, PA imaging is ideal to pair with PTT to develop optical theranostics.^84^ However, the endogenous contrast is limited to hemoglobin, lipids, and melanin.[[qv: 83b]] Therefore, the full utilization of PA imaging in biological and medical fields heavily rely on the development of efficient PA probes.

### 4.1 ROS

**Figure 11:**
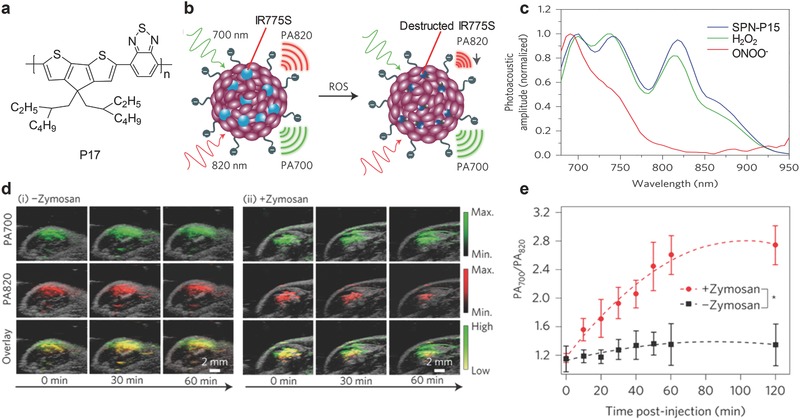
SPN‐based activatable probe for PA imaging of ROS. a) The chemical structure of P17 used for preparation of the SPN‐P17 probe for PA imaging of ROS. b) Illustration of the mechanism of detection of ROS. c) PA spectra of the SPN‐P17 probe in the absence and presence of ROS. d) PA/ultrasound overlaid images of saline‐treated (i) and zymosan‐treated (ii) regions in the thigh of living mice. The SPN‐P17 probe was intramuscularly injected into the thigh 20 min after zymosan treatment. e) The ratiometric PA signals (PA_700_/PA_820_) as a function of time post‐injection of the SPN‐P17 probe. *Statistically significant difference in PA_700_/PA_820_ between zymosan‐treated and saline‐treated mice at all time points starting from 10 min (*P* < 0.05). Reproduced with permission.[[qv: 17b]] Copyright 2014, Macmillan Publishers Limited, part of Springer Nature.

A NIR light absorbing SP, poly(cyclopentadithiophene‐*alt*‐benzothiadiazole) (P17, **Figure** [qv: **11**]a), was doped with an organic NIR dye IR775S (Figure 8a) to form the SPN‐based probe for in vivo PA imaging of ROS.[[qv: 17b]] The probe emitted strong PA signals with the maxima at 700, 735, and 820 nm. In the presence of ROS, the peaks at 735 and 820 nm corresponding to IR775S decreased because of the destruction of IR775S, while the peak at 700 nm remained unchanged (Figure 11b,c). Thus, the ratiometric PA signals (PA_700_/PA_820_) were generated, showing specificity towards certain ROS including ONOO^−^ and ClO^−^. The capability of the SPN‐P17 probe for in vivo imaging of ROS was validated in a murine model with acute oedema induced by zymosan. After treatment of zymosan, the PA signals of the SPN‐P17 probe significantly decreased at 820 nm without obvious change at 700 nm (Figure 11d), and thus PA_700_/PA_820_ gradually increased. (Figure 11e). These data indicated that the SPN‐P17 was activated by ROS produced in inflammatory region.

### 4.2 pH

**Figure 12:**
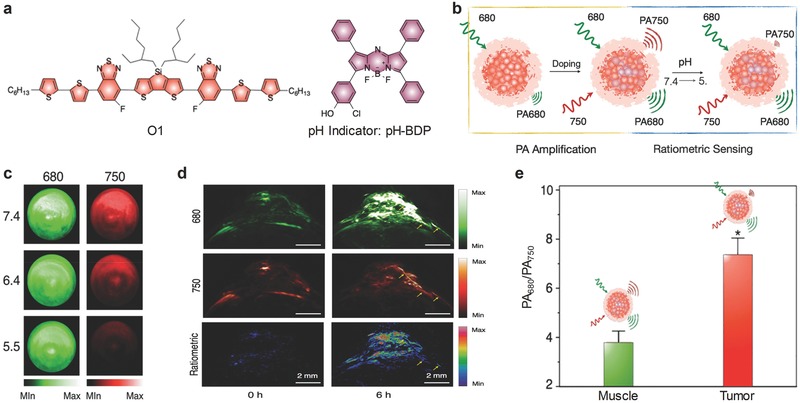
PA imaging used in pH detection. a) The chemical structures of the semiconducting oligomer (O1) and the organic dye (pH‐BDP) used for preparation of the SON‐O1 probe for PA imaging of pH. b) Illustration of the mechanism for detection of pH. c) PA images of the SON‐O1 solution at pH 7.4, 6.4, and 5.5. A pulsed laser was turned to 680 and 750 nm for ratiometric PA imaging. d) PA and ratiometric images (PA_680_/PA_750_) of a subcutaneous HeLa tumor in a nude mouse before and 6 h after systemic administration of the SON‐O1 probe. The yellow arrows indicated one of the blood vessels of the tumor. e) Ratiometric PA signals (PA_680_/PA_750_) of the muscle and the tumor with local administration of the SON‐O1 probe. *Statistically significant difference in PA_680_/PA_750_ between the muscle and the tumor (*P* < 0.001, *n* = 3). Reproduced with permission.[[qv: 17e]] Copyright 2016, WILEY‐VCH Verlag GmbH & Co. KGaA, Weinheim.

Pu's group proposed a facile nanoengineering approach to develop activatable PA probes based on semiconducting oligomer nanoparticles (SONs) for pH imaging.[[qv: 17e]] Because of the low molecular weight of semiconducting oligomer (SO1, Figure 12a), the resulted SONs were ultrasmall with diameters of ≈10 nm. The sensing dye, pH‐BDP (**Figure** [qv: **12**]a), was responsive to pH, and well paired with SO1 to favor efficient PET and in turn promoted non‐radiative thermal deactivation. This led to amplified PA signals for the probe (Figure 12b). With increasing acidity, the PA signals at 750 nm decreased but those at 680 nm remained approximately unchanged (Figure 12c). Thus, the ratiometric PA signals (PA_680_/PA_750_) could quantify pH, showing a good linearity between 5.5 and 7.4. Because of the good biodistribution, the SON‐based probe permitted in vivo ratiometric PA imaging of pH in the tumors of living mice after systemic administration (Figure 12d). Compared with normal tissue, the significantly stronger signals in the tumor proved the effectiveness of the SON‐O1 probe in sensing subtle pH variations in a physiological environment (Figure 12e).

## 5 Conclusion

As a new generation of purely organic photonic nanoagents, SPNs have been proven to be effective in the sensing and imaging of small molecules and biomacromolecules both in vitro and in vivo. The structural diversity of SPs makes it feasible to transform them into smart activatable probes with different imaging modalities, including photoluminescence, chemiluminescence and PA imaging. SPN‐based activatable probes are usually developed by incorporation of sensing or target‐responsive components. In the presence of the target of interest, the responsive part changes its structure or induces the changes of interparticle/intraparticle distances, resulting in the alteration of optical signals. The SP within the probes generally acts as the internal reference, as SPs have good photostability and biological inertness. Particularly, the light‐harvesting properties of SPNs allows them to act as the energy donors in FRET processes to amplify the optical signals and subsequently reduce the limit of detection. With these merits, SPNs serve as a versatile nanoplatform to construct photonic activatable probes with high sensitivity.

Although SPN‐based activatable probes hold great promise in sensing and molecular imaging, challenges are present to further advance their applications in life science. The long‐term safety has not reached a definite conclusion despite the recent studies illustrating that SPNs have no acute toxicity in living mice.[[qv: 17d]] To solve this concern, biodegradation and fast clearance are desired for these organic activatable probes. Fast clearance can be achieved by reducing their sizes below 5 nm so that urinary excretion can occur, while biodegradation is more challenging in terms of chemistry. The biodegradable amphiphilic polymers such as poly(lactide‐*co*‐glycolide) (PLGA) based polymers can be used to encapsulate SPs, but how to make SPs biodegradable without compromising their optical properties remain elusive.

Application of SPNs in biomedical science and engineering is still at the early stage. However, the flexibility and excellent optical properties of SPNs will permit integration of other functionality into them and allow for applications beyond sensing and imaging.
